# Supplementary material for: Feedback inhibition of AMT1 NH4+-transporters mediated by CIPK15 kinase
Source: BMC Biol. 2020 Dec 14;18:196. doi: 10.1186/s12915-020-00934-w (PMC7737296; doi:10.1186/s12915-020-00934-w)
Supplement: Supplementary file 1 — Additional file 1: Figure S1. Screen for CIPK effects on AMT1;1 activity in Xenopus oocytes. Figure S2. The activity of AMT1;1 co-expressed with CIPKs or CBL in Xenopus oocytes. Figure S3. Protein gel blots for AMT1;1 protein level in Xenopus oocytes as control for Fig. 1. Figure. S4. Alignment of the TMH XI and C terminus of five members of the AMT1 family. Figure S5. CIPK19 has no effect on AmTryoshka1;3 LS-F138I activity in yeast. Figure S6. Mating-based split-ubiquitin assay, controls for Fig. 4a. Figure S7. CIPK15 can interact with AMT1;1, AMT1;2, and AMT1;3 in yeast. Figure S8. Split-fluorescent protein interaction assay for AMT1;1 and CIPKs in Nicotiana benthamiana leaves. Figure S9. Arabidopsis T-DNA insertion mutants of cipk15-1 and cipk15-2. Figure S10. Protein gel blots for AMT1;1 protein and AMT-P phosphorylation levels in wild-type and cipk15 mutant plants under half-strength MS medium. Figure S11. Primary root length of control (wild-type) and qko mutant on half-strength MS medium containing NH4Cl, KNO3, or MeA. Figure S12. Primary root length of Col-0, qko, and cipk15 mutant plants on media containing KNO3 as sole nitrogen source. Figure S13. cbl4 and cipk19 mutants do not show ammonium hypersensitivity. Figure S14. CIPK23 mRNA accumulated by NH4+. [file 12915_2020_934_MOESM1_ESM.docx]

**BMC Biology Supporting Information:**

**Article title**:

**Feedback inhibition of AMT1 NH_4_^+^-transporters mediated by CIPK15 kinase**

**Authors:**

**Hui-Yu Chen^1^, Yen-Ning Chen^1^, Hung-Yu Wang^1^, Zong-Ta Liu^1^, Wolf B. Frommer^2,3^ & Cheng-Hsun Ho^1,^***

**Additional file 1: Figures**


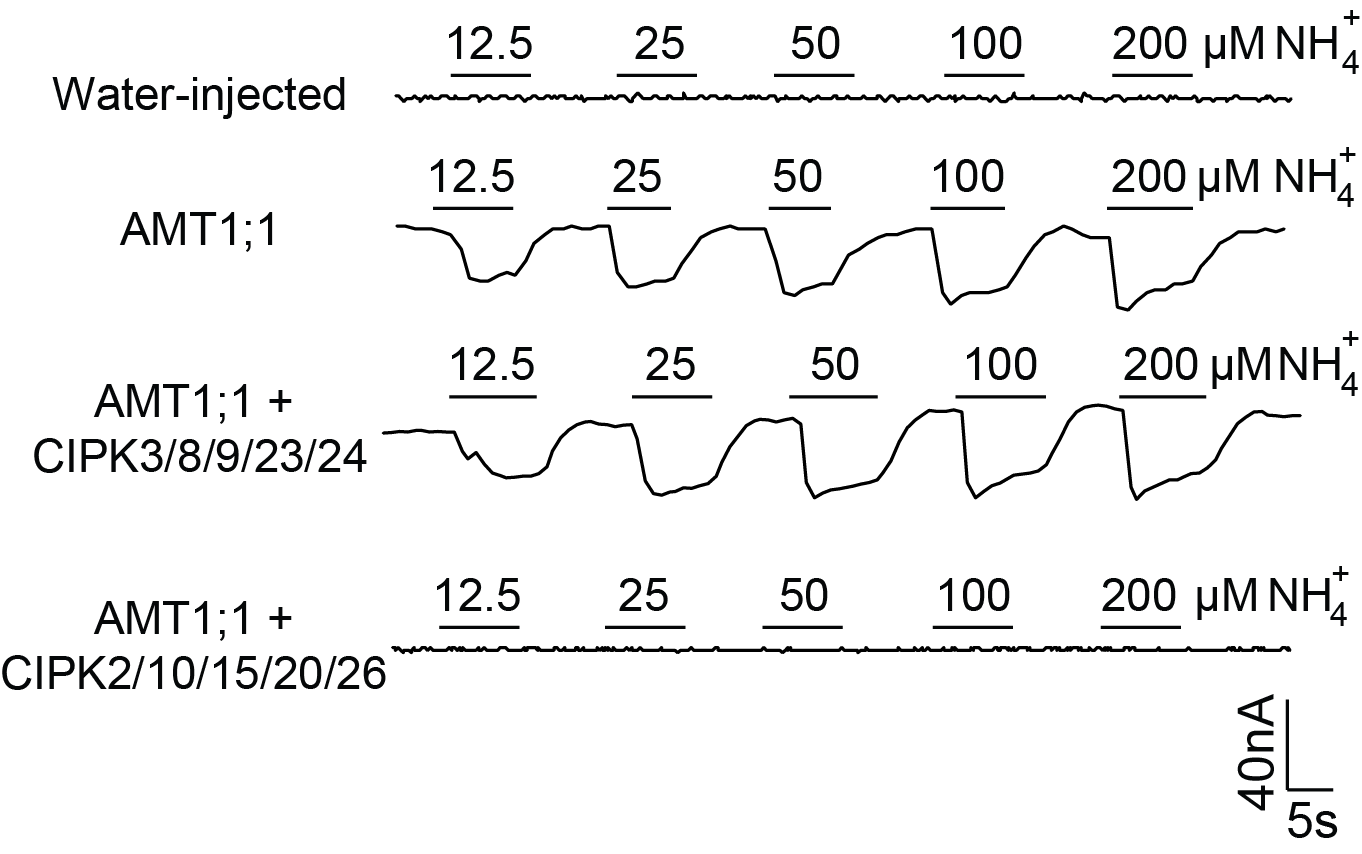


**Figure S1. Screen for CIPK effects on AMT1;1 activity in *Xenopus* oocytes.** Different combinations of CIPKs were analyzed for effects on AMT1:1 transport activity. Shown are currents recorded in *Xenopus* oocytes injected with water (control), AMT1;1 alone, AMT1;1 + CIPK3/8/9/23/24, or AMT1;1 + CIPK2/10/15/20/26 (50 ng for each gene). cRNAs of AMT1;1 and CIPKs or water as control were injected into oocytes, subjected to TVEC and perfused with NH_4_Cl at the indicated concentrations. Oocytes were clamped at −120 mV (similar results were obtained in multiple independent experiments using different batches of oocytes). The results indicate that either one or several CIPKs in combination from the mixture of CIPK2, 10, 15, 20 and 26 may have impaired AMT1;1 activity, while CIPK3, 8, 9, 23 and 24 had no major impact on AMT1;1 activity.


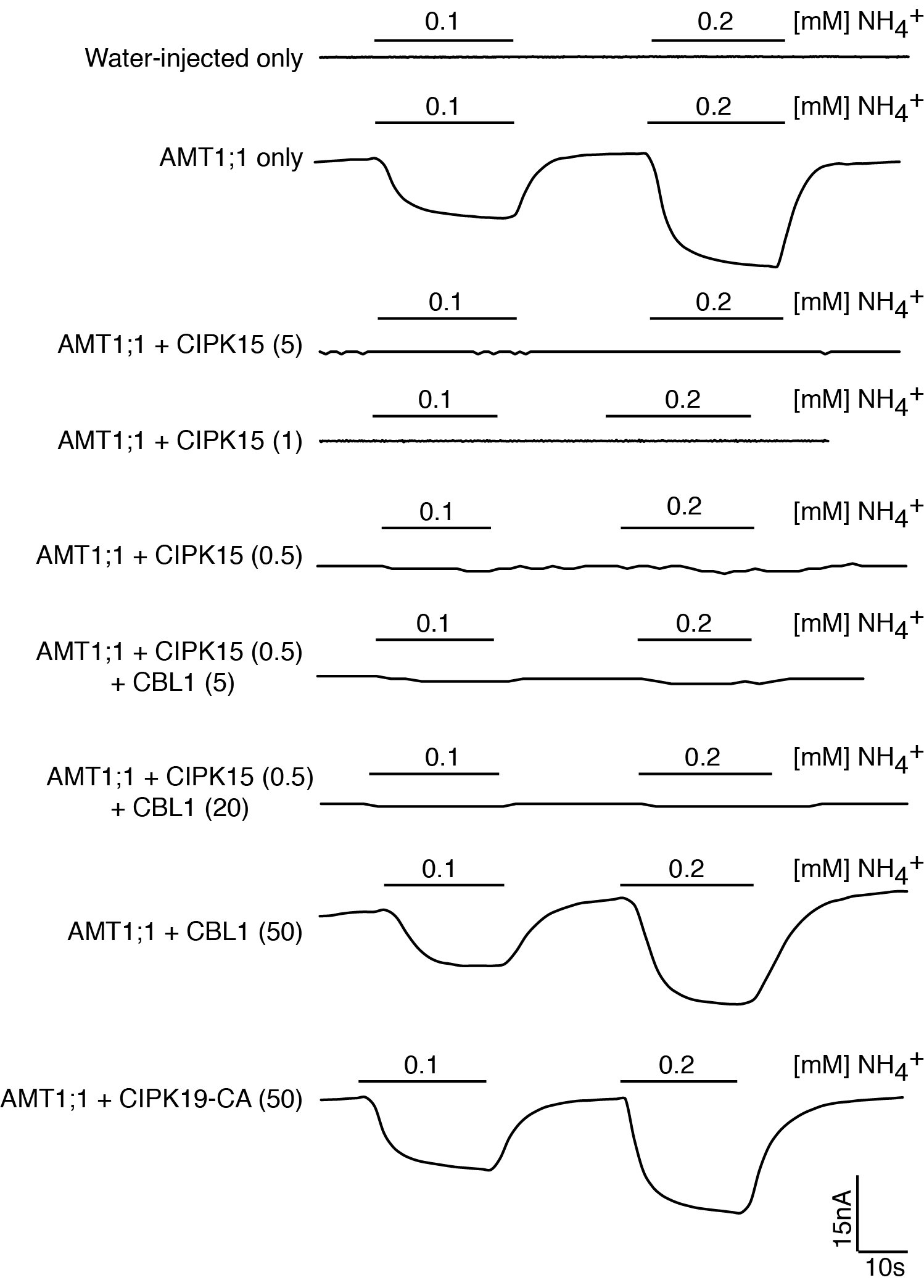


**Figure S2. The activity of AMT1;1 co-expressed with CIPKs or CBL in *Xenopus* oocytes.** Five nanograms of cRNA of AMT1;1, and different nanograms of cRNAs of CBL1, CIPK15, CIPK19-CA as indicated in the figure, or control (H_2_O) were injected into oocytes. AMT1;1 activity was considerably reduced when co-injected with different amounts of *CIPK15* cRNA, whereas, the AMT1;1 activity was not affected when co-expressed with CBL1 and CIPK19-CA in *Xenopus* oocytes. Currents recorded in single oocytes injected with water, AMT1;1, AMT1;1 + CBL1, AMT1;1 + CIPK15, AMT1;1 + CBL1 + CIPK15, or AMT1;1 + CIPK19-CA. Oocytes were perfused with square pulses of 0.1 mM and 0.2 mM NH_4_Cl as indicated, respectively. Oocytes were clamped at −120 mV (independent data from three different oocytes were recorded from three different batches with comparable results).


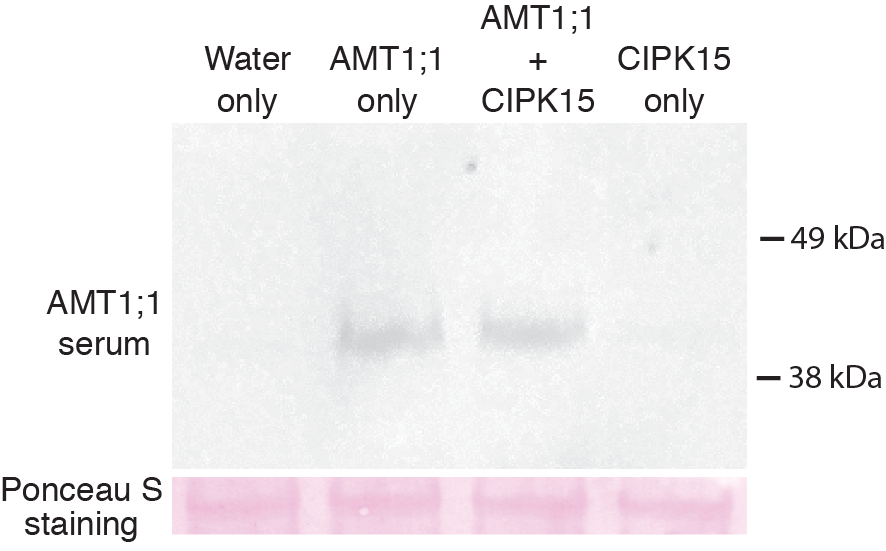


**Figure S3. Protein gel blots for AMT1;1 protein level in *Xenopus* oocytes as control for Figure 1.** Immunodetection of AMT1 protein using affinity-purified peptide antisera against a domain in the unphosphorylated cytosolic C-terminus (marked AMT1;1) [25]. 10% SDS PAGE. AMT1;1 protein levels were detected in membrane fractions of *Xenopus* oocytes, which were injected with water only, cRNA of AMT1;1 only (50 ng), AMT1;1 (50 ng) + CIPK15 (50 ng), and CIPK15 (50 ng) only. Ponceau S staining of filters before transfer served as the loading control. Comparable results were obtained in three independent experiments.


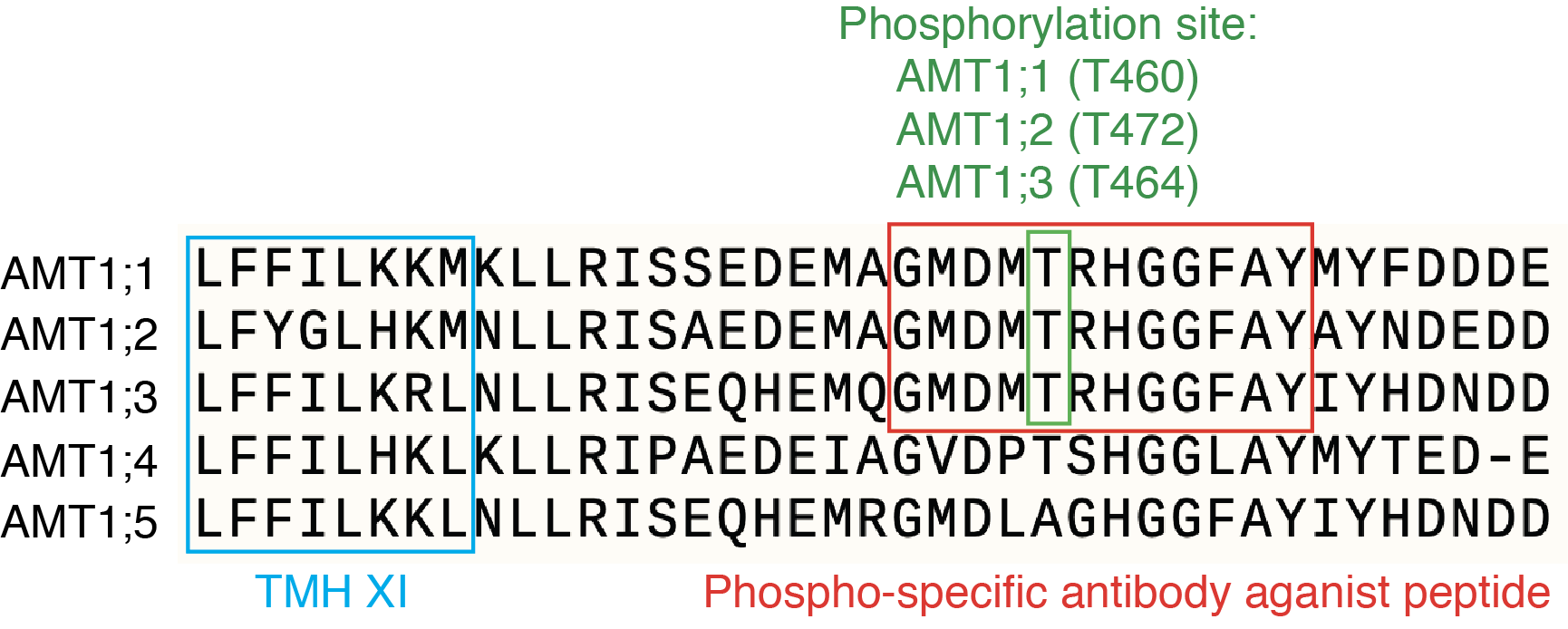


**Figure. S4. Alignment of the TMH XI and C terminus of five members of the AMT1 family.** The C-terminal part of TMH XI is boxed in blue and the peptide sequence, specifically against the AMT1-P phospho-antiserum, is boxed in red. Phosphorylation sites of AMTs recognized by AMT1-P are boxed in green.


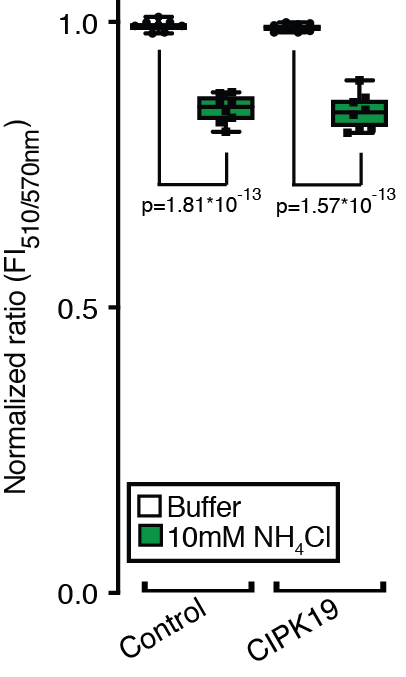


**Figure S5.** **CIPK19 has no effect on AmTryoshka1;3 LS-F138I activity in yeast.** Unlike CIPK15, CIPK19 did not impair NH_4_^+^-triggered AmTryoshka1;3 LS-F138I [15] responses in yeast. Amtryoshka1;3 LS-F138I was co-expressed with control (vector only) and CIPK19. As described in Fig. 2, results of normalized fluorescence ratio (normalized to buffer control=1, *λ* _exc_ 440 nm, ratio= FI_510nm_/_570nm_) after addition of NH_4_Cl are represented by box and whiskers (mean ± SE, n=8). Center lines show the medians; box limits indicate the 25th and 75th percentiles as determined by Prism software; whiskers extend 1.5 times the interquartile range from the 25th and 75th percentiles, outliers are represented by dots. p, significant change as shown in the figure (Two-Way ANOVA followed by Tukey’s post-test).


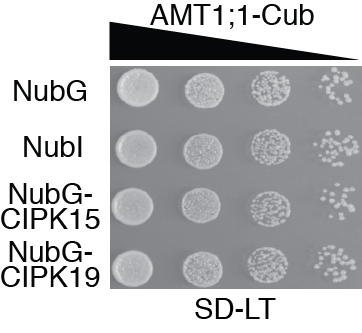


**Figure S6. Mating-based split-ubiquitin assay, controls for Figure 4a.** Growth assay in non-selective conditions on SD- Leu/Trp (LT) medium with identical cultures as shown in Fig. 4a and performed in parallel. Plasmids expressing AMT1;1, CIPK15, and CIPK19 were expressed in yeast. After overnight growth, colonies were serially diluted fourfold and grown for 2 days on SD-LT medium. As described in Fig. 4, full-length AMT1;1-Cub-PLV as bait and NubG, NubI, and NubG-full-length CIPK15 or CIPK19 as prey. No difference in growth of yeast was observed for yeast cells expressing AMT1;1-Cub control plasmids or NubG-CIPK15 or -CIPK19 expressing cells. Comparable results were obtained in three independent experiments.


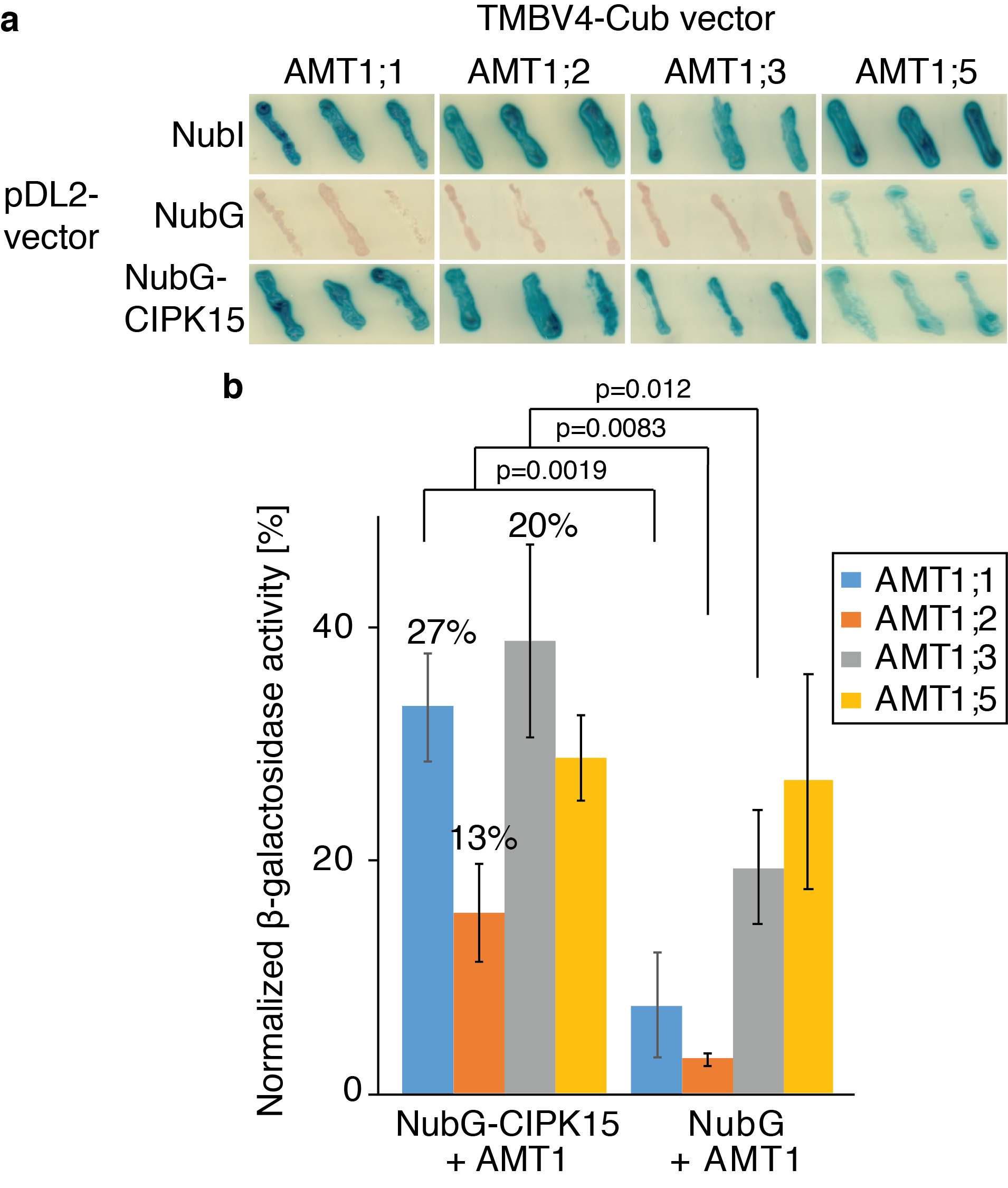


**Figure S7. CIPK15 can interact with AMT1;1, AMT1;2, and AMT1;3 in yeast.** Yeast mating-based split-ubiquitin assay for CIPK15 interaction with AMT1;1, AMT1;2, AMT1;3, or AMT1;5. Plasmids expressing AMT1;1, AMT1;2, AMT1;3, AMT1;5 with CIPK15 were expressed in yeast. Interactions were monitored using qualitative and quantitative ß-galactosidase activity assays. (**a**) Blue X-gal staining on plates, and (**b**) ß-galactosidase assay. Expression of pDL2-NubG was used as a negative control; pDL2*-*NubI was used as a positive control. For quantitative ß-galactosidase assays, cells were grown to an OD_600_ = 0.8. Activity was measured as described in the Materials and Methods section and normalized to NubI as 100% (mean ± SE). The numbers above the error bars represent the results of interaction strength after subtracting the NubG negative control (NubG + AMT1). Values from five samples were averaged for each bait-prey combination. p, significant change for ß-galactosidase activity compared to vector only (Two-Way ANOVA followed by Tukey’s post-test).


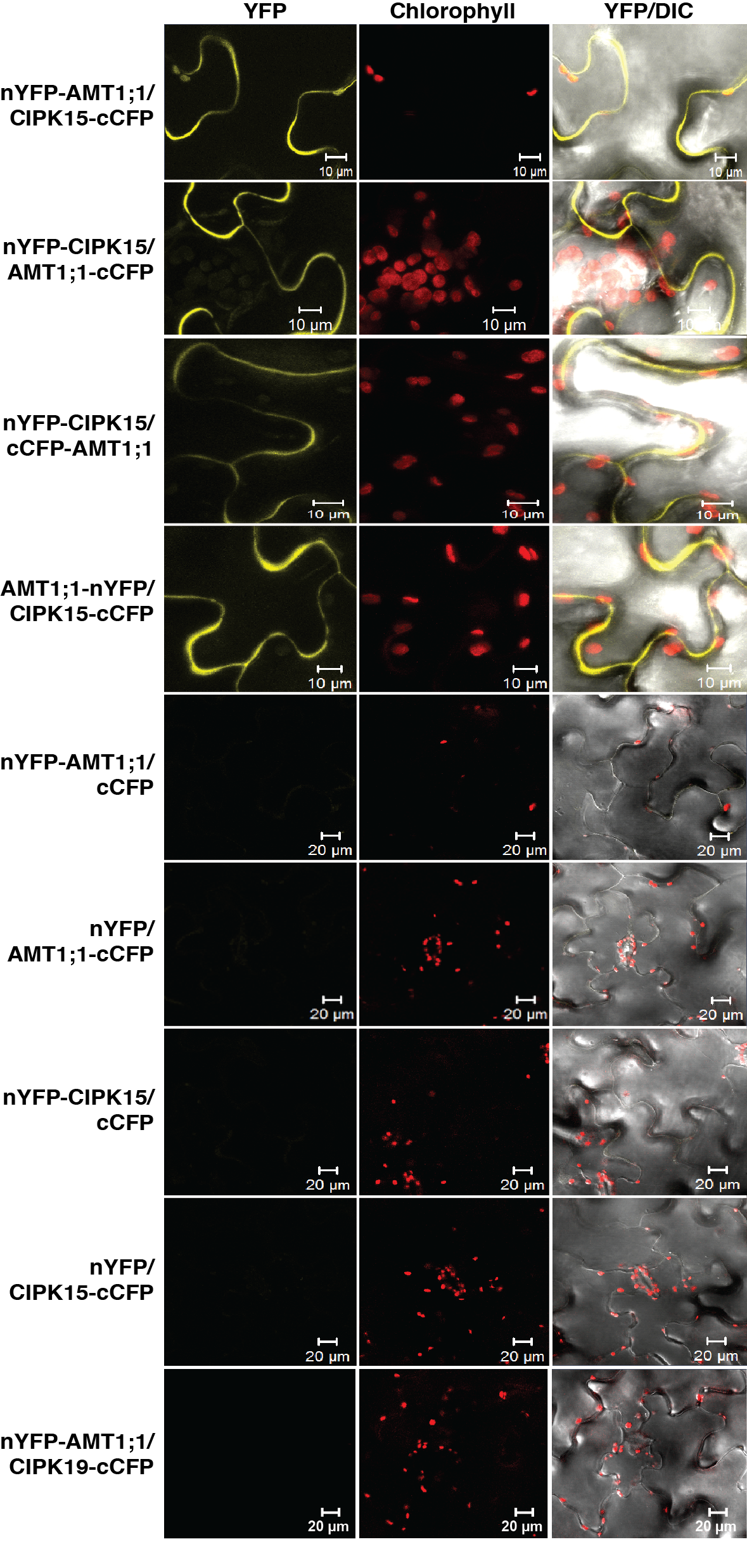


**Figure S8. Split-fluorescent protein interaction assay for AMT1;1 and CIPKs in *Nicotiana benthamiana* leaves.** Split-fluorescent protein interaction assay as described in [52]. Different combinations of reconstitution of YFP fluorescence for AMT1;1, CIPK15, and CIPK19 (nYFP-AMT1;1 + cCFP, nYFP + AMT1;1-cCFP, nYFP-CIPK15 + cCFP, nYFP + CIPK15-cCFP, nYFP-AMT1;1 + CIPK15-cCFP, nYFP-CIPK15 + AMT1;1-cCFP, nYFP-CIPK15 + cCFP-AMT1;1, AMT1;1-nYFP + CIPK15-cCFP, nYFP-AMT1;1 + CIPK19-cCFP) are shown. Chloroplast panels show chlorophyll autofluorescence; YFP/DIC, merged image of fluorescence, chloroplast, and brightfield images.

**
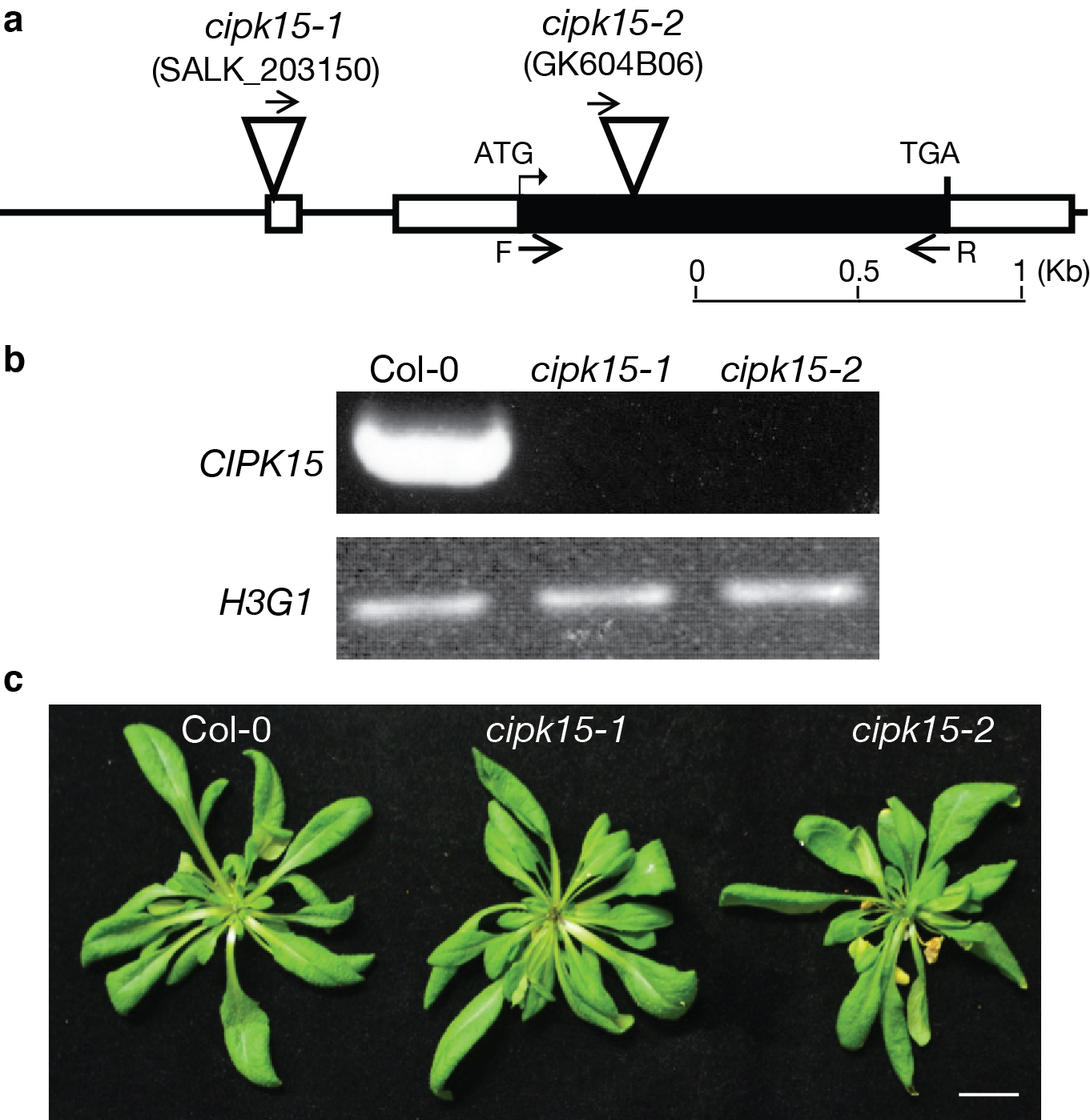
**

**Figure S9. Arabidopsis T-DNA insertion mutants of *cipk15-1* and *cipk15-2*.** (**a**) Schematic map of the *cipk15-1* and *cipk15-2* mutants showing the positions of T-DNA insertions in *CIPK15*. F, *CIPK15* forward primer; R, CIPK15 reverse primer. Gene transcribed from left to right. Solid black box: exon, white boxes 5′-UTR and 3′-UTR, respectively. *cipk15-1* carries an insertion in the 5′-UTR, *cipk15-2* carries an insertion in the coding region. (**b**) RT-PCR analysis of *CIPK15* transcript levels in *cipk15-1* and *cipk15-2* mutant lines. H3G1 (At4g40040) was used as the loading control. None of the mutant lines showed detectable amounts of *CIPK15* mRNA, indicating that both are knockout mutants. Note that *CIPK15* contains only a single intron in the 5′-UTR. (**c**) Col-0 and *cipk15* mutants showed no obvious growth differences when grown in soil or axenically in MS medium. Representative images of rosette leaves of Col-0 and *cipk15* mutants. Plants were grown in soil under MS medium in a 16/8 h light/dark period at 22ºC for 17 days. Scale bar: 1cm.

**
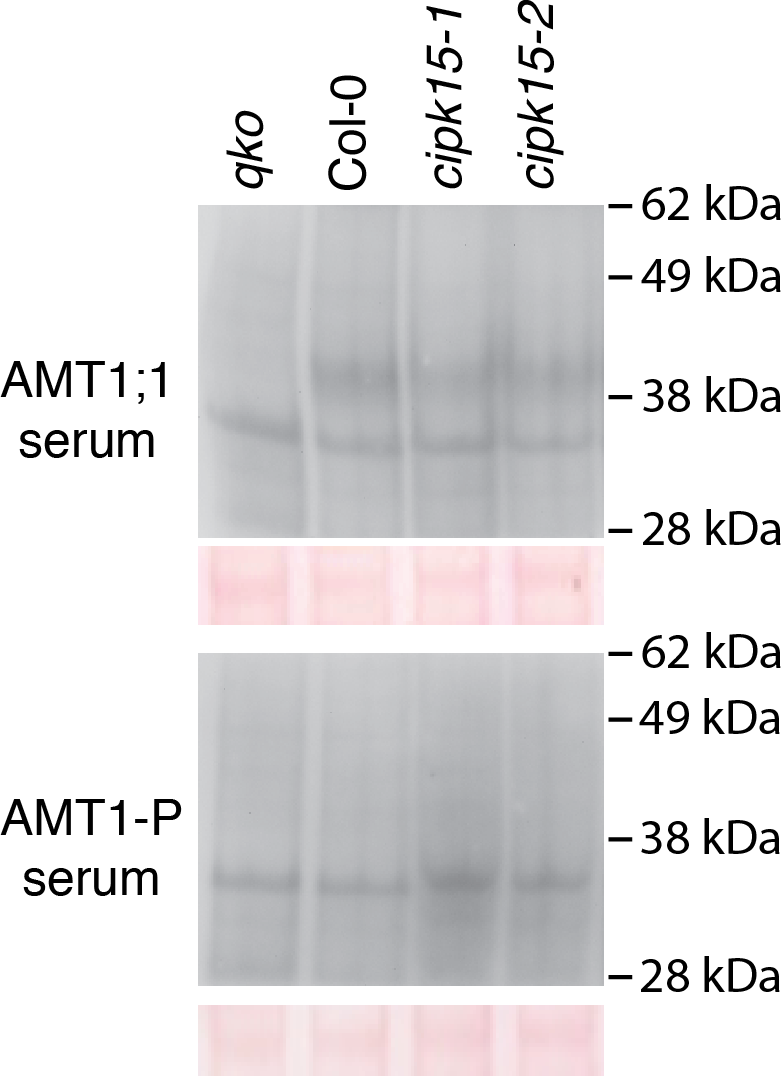
**

**Figure S10. Protein gel blots for AMT1;1 protein and AMT-P phosphorylation levels in wild-type and *cipk15* mutant plants under half-strength MS medium.** Immunodetection of AMT1 protein using affinity-purified peptide antisera against a domain in the unphosphorylated cytosolic C-terminus (marked AMT1;1) and affinity-purified antisera against the phosphorylated cytosolic C-terminus (marked AMT1-P) [25]. 10% SDS PAGE. AMT1;1 protein and AMT-P levels were detected in roots of plants grown on half-strength MS medium for 7 days as described [25]. Ponceau S staining of filters before transfer served as the loading control. Comparable results were obtained in three independent experiments.

**
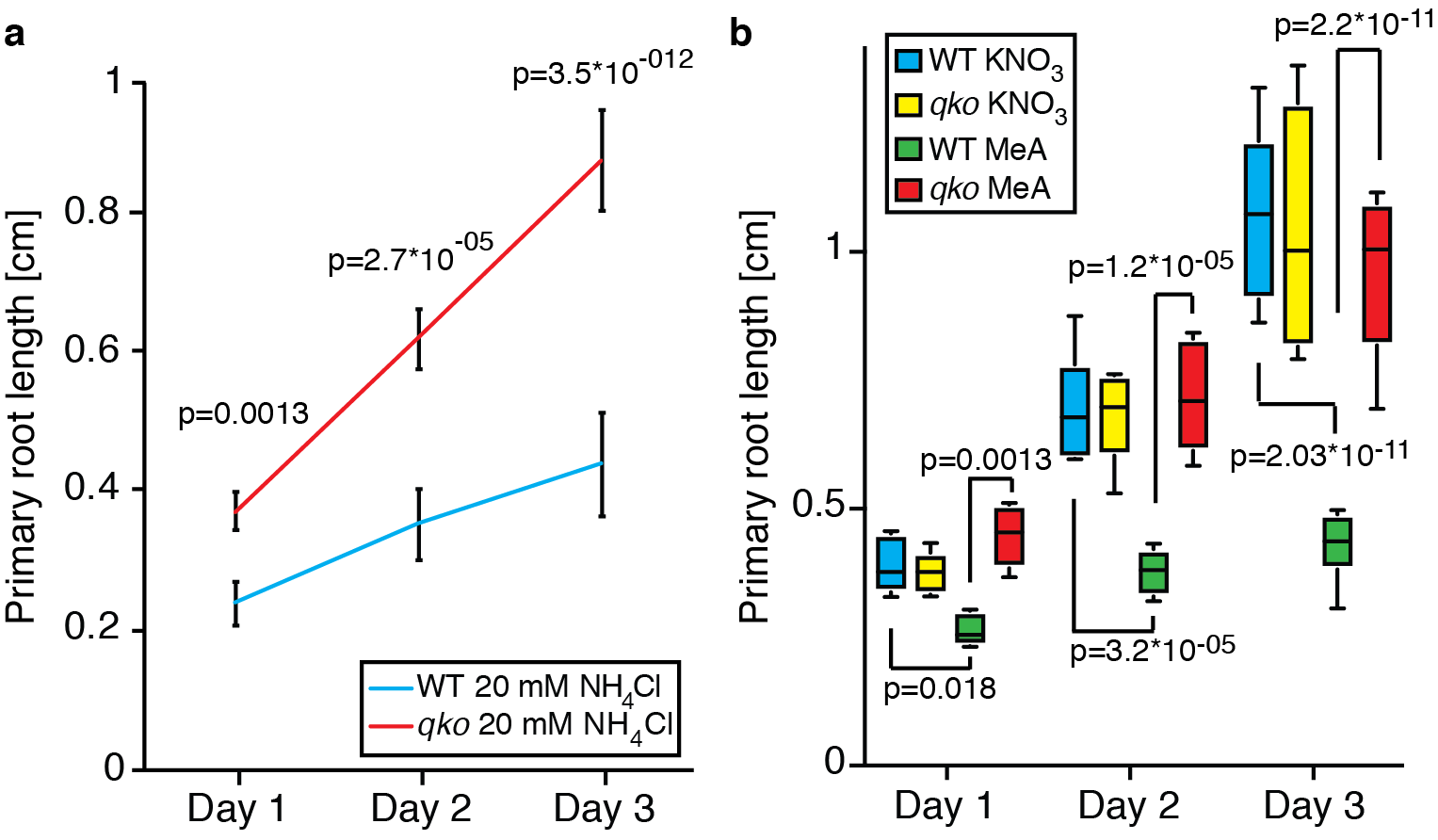
**

**Figure S11. Primary root length of control (wild-type) and *qko* mutant on half-strength MS medium containing NH_4_Cl, KNO_3_, or MeA.** Seedlings were grown on half-strength MS medium containing NH_4_Cl, KNO_3_ or on MeA. Primary root length was measured in wild-type (Col-0) and *qko* mutant. (**a** and **b**) Primary root length 1, 2, or 3 days after transferring wild-type and *qko* mutant plants to 20 mM NH_4_Cl, MeA or KNO_3_. Data are mean ± SE; n ≥5. p, significant change between wild-type and *qko* mutant (Two-Way ANOVA followed by Tukey’s post-test).

**
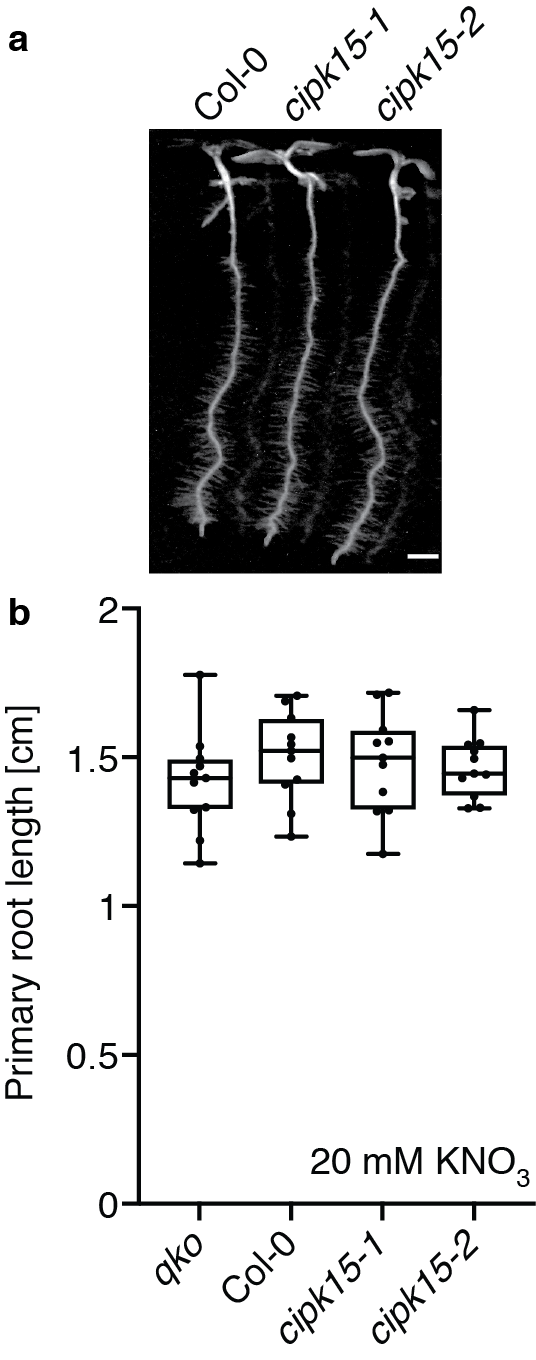
**

**Figure S12. Primary root length of Col-0, *qko*, and *cipk15* mutant plants on media containing KNO_3_ as sole nitrogen source.** Representative images (**a**) and quantification results of primary root length of plants grown on plates containing 20 mM KNO_3_. Scale bar: 0.1 cm. Primary root length in wild-type (Col-0), *qko* mutant and *cipk15* mutant plants on 20 mM KNO_3_ (n =11) presented as box and whiskers. Center lines show the medians; box limits indicate the 25th and 75th percentiles as determined by Prism software; whiskers extend 1.5 times the interquartile range from the 25th and 75th percentiles, outliers are represented by dots. Data are mean ± SE. No significant change between wild-type, *qko*, and *cipk15* mutants (Two-Way ANOVA followed by Tukey’s post-test).

**
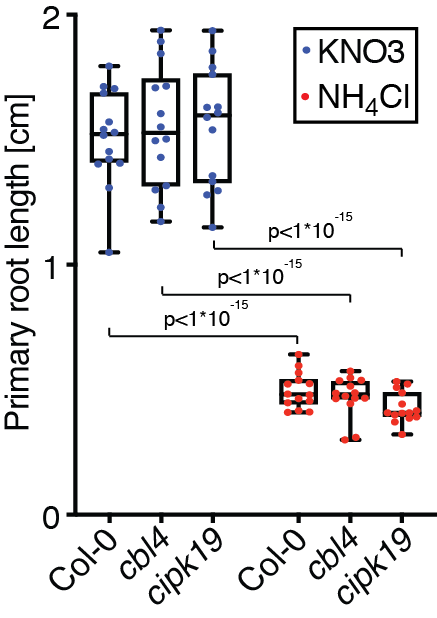
**

**Figure S13. *cbl4 and cipk19* mutants do not show ammonium hypersensitivity.** Primary root length of control (Col-0), *cbl4* (At5g24270, SALK_113101) [48] and *cipk19* (At5g45810, SALK_044735) [47] knockout mutant plants on half-strength MS medium containing NH_4_Cl and KNO_3_. Seedlings were germinated and grown on half-strength MS medium containing 5 mM KNO_3_ for 3 days, and then, primary root length of wild-type (Col-0) and mutants were measured after transferral to 20 mM NH_4_Cl or KNO_3_ conditions for 5 days and presented as box and whiskers. Data are mean ± SE; n=14. p, significant difference between NH_4_Cl and KNO_3_ (Two-Way ANOVA followed by Tukey’s post-test). The center lines show the medians; box limits indicate the 25th and 75th percentiles as determined by Prism software; whiskers extend 1.5 times the interquartile range from the 25th and 75th percentiles.


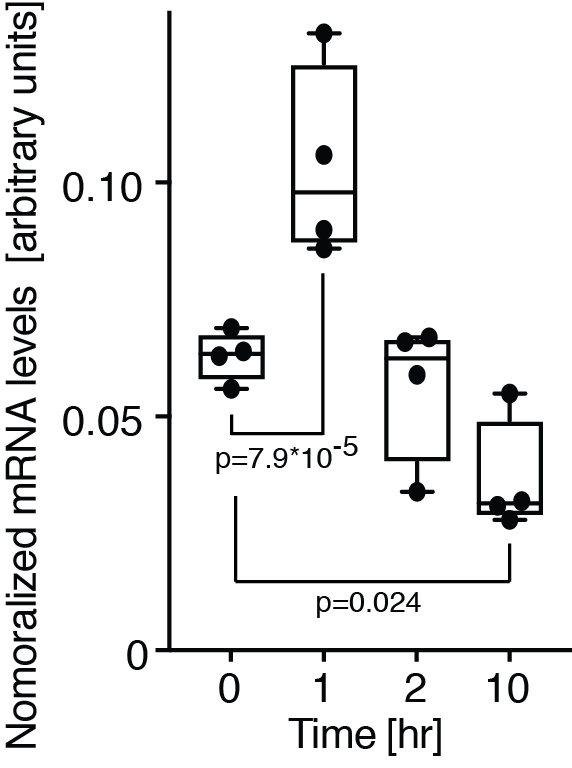


**Figure S14. *CIPK23* mRNA accumulated by NH_4_^+^.** qRT-PCR analyses of *CIPK23* mRNA levels in roots after over 10 h after addition of 1 mM NH_4_^+^ in Col-0. Levels were normalized to *UBQ10* [mean ± SE for four independent experiments (each experiment n >50, total n >200)]. p, significant change in mRNA levels of *CIPK23* at 1, 2, and 10 h compared to at 0 h (Two-Way ANOVA followed by Tukey’s post-test).
